# Multiple Antibiotic-Resistant, Extended Spectrum-β-Lactamase (ESBL)-Producing Enterobacteria in Fresh Seafood

**DOI:** 10.3390/microorganisms5030053

**Published:** 2017-08-30

**Authors:** Asem Sanjit Singh, Manjusha Lekshmi, Sreepriya Prakasan, Binaya Bhusan Nayak, Sanath Kumar

**Affiliations:** Quality Control Laboratory, Post Harvest Technology Department, Central Institute of Fisheries Education (CIFE), Mumbai 400061, India; sanjitasem21@gmail.com (A.S.S.); manjusha@cife.edu.in (M.L.); sreepriya.phtpa501@cife.edu.in (S.P.); nayakbb@cife.edu.in (B.B.N.)

**Keywords:** seafood, enterobacteria, ESBL, *bla*_NDM_, antibiotic resistance

## Abstract

Members of the family *Enterobacteriaceae* include several human pathogens that can be acquired through contaminated food and water. In this study, the incidence of extended spectrum β-lactamase (ESBL)-producing enterobacteria was investigated in fresh seafood sold in retail markets. The ESBL-positive phenotype was detected in 169 (78.60%) isolates, with *Escherichia coli* being the predominant species (53), followed by *Klebsiella oxytoca* (27), and *K. pneumoniae* (23). More than 90% of the isolates were resistant to third generation cephalosporins, cefotaxime, ceftazidime, and cefpodoxime. Sixty-five percent of the isolates were resistant to the monobactam drug aztreonam, 40.82% to ertapenem, and 31.36% to meropenem. Resistance to at least five antibiotics was observed in 38.46% of the isolates. Polymerase Chain Reaction (PCR) analysis of ESBL-encoding genes detected *bla*_CTX_, *bla*_SHV_, and *bla*_TEM_ genes in 76.92%, 63.3%, and 44.37% of the isolates, respectively. Multiple ESBL genes were detected in majority of the isolates. The recently discovered New Delhi metallo-β-lactamase gene (*bla*_NDM-1_) was detected in two ESBL^+^ isolates. Our study shows that secondary contamination of fresh seafood with enteric bacteria resistant to multiple antibiotics may implicate seafood as a potential carrier of antibiotic resistant bacteria and emphasizes an urgent need to prevent environmental contamination and dissemination of such bacteria.

## 1. Introduction

Members of the family *Enterobacteriaceae*, which include important food-borne pathogens such as *Salmonella enterica* and *Escherichia coli*, are known to cause diverse types of infections ranging from wound infection to meningitis, and are also known agents of nosocomial infections. In recent years, the chemotherapeutic options for enterobacteria are becoming severely constricted owing to the development of resistance to multiple antibiotics, the most notable among these being resistance to β-lactam group of antibiotics such as cephalosporins and carbapenems [1]. Cephalosporin resistance is accomplished by the production of one or more types of β-lactamases called extended spectrum-β-lactamases (ESBLs) [2,3]. ESBL-producing Gram-negative bacteria have become a severe challenge to chemotherapy [4,5]. ESBLs are classified into several groups, the prominent among them being TEM, SHV, and CTX-M types [6]. ESBLs confer resistance to third generation cephalosporins (e.g., cefotaxime, ceftazidime) and monobactams (e.g., aztreonam), but cannot hydrolyze cephamycins (cefoxitin) or carbapenems (imipenem), and are inhibited by β-lactamase inhibitors such as clavulanic acid [7]. Carbapenems are the antibiotics of choice against ESBL-producing bacteria, but the occurrence of carbapenem resistant enterobacteria (CRE) producing plasmid-encoded metallo-β-lactamases with carbapenemase activity have emerged worldwide [8,9]. In 2008, a new β-lactamase, the New Delhi metallo-β-lactamase (*bla*_NDM-1_) capable of hydrolyzing all β-lactams with the exception of aztreonam, was identified in a clinical isolate of *Klebsiella pneumoniae* [10]. Subsequently, the NDM-1 producing enterobacteria were isolated from different parts of the world and their rapid dissemination has become a global concern [11,12]. Studies from India and other countries have reported the occurrence of *bla*_NDM_-harboring bacteria in the environment [13,14].

The presence of antibiotic-resistant bacteria in seafood is not only a threat to human health, but also can result in the transfer of resistant determinants to other clinically important bacteria. Seafood is relatively free of human pathogens, except for vibrios which are natural contaminants of seafood from the marine environment. The occurrence of human enteric pathogens such as *Escherichia coli* and *Salmonella enterica* in seafood is due to the contamination of water bodies from where the fish are harvested or contaminations occurring at various stages of handling after harvest [15]. Studies from India have shown the occurrence of enteric pathogens in seafood [16,17,18]. The threat due to the presence of enteric pathogens in seafood is more confounding when such bacteria are multi-drug resistant (MDR). The prevalence of MDR bacteria in seafood has been reported in many studies in the recent past [19,20,21,22,23]. The antibiotic resistance patterns of clinical isolates of enteric bacteria is alarmingly high in India and with the discovery of NDM-producing enteric bacteria, the concerns on the rapid spread of such bacteria via food chain and water is increasing [24,25]. We recently reported the isolation of *bla*_NDM5_-harboring *Escherichia coli* from fresh seafood in Mumbai, India which prompted further investigation into the incidence of antibiotic resistant bacteria in fresh seafood in this region [26]. Since the coastal water bodies of this study region are subjected to constant pollution from anthropogenic sources, the present work was initiated with the aim of studying the prevalence of antibiotic-resistant enterobacteria in seafood.

In the study reported here, fresh seafood from landing centers and retail markets were analyzed for the presence of ESBL-producing enterobacteria. The results suggest the occurrence of diverse ESBL-producing enterobacteria resistant to multiple drugs including cephalosporins, carbapenems, and fluoroquinolones. The isolates were tested for the common genes responsible for the ESBL^+^ phenotype.

## 2. Materials and Methods

### 2.1. Sample Collection, Isolation, and Identification of Enterobacteria from Seafood

Fresh seafood were collected from the retail markets of Western Mumbai between August 2013 and April 2014. Nineteen samples collected during the period consisted of fish (14), shrimps (3), clam (1), and squid (1). The samples were stored in sterile collection bags containing ice, transported to the laboratory immediately and processed for the isolation of enterobacteria by selective enrichment followed by selective plating. Twenty-five grams of fish or shellfish was homogenized with 225 mL of enterobacteria enrichment (EE) broth (Mossel) (Hi-Media, Mumbai, India) in a sterile bag and incubated statically for 16 h at 37 °C. Two-loopfuls from the enrichment broth were streaked on MacConkey agar (Hi-Media, Mumbai, India) before and after incubation, and the colonies were purified on Luria Bertani (LB) agar. Oxidase-negative isolates were subjected to a series of biochemical tests for the identification of different species of enterobacteria [27]. In cases of ambiguity, the species identity of the isolates was confirmed by sequencing of the 16SrRNA genes [28].

### 2.2. Antibiotic Susceptibility Tests

The preliminary screening for ESBL production was done by spotting on HiChrome ESBL agar (Hi-Media, Mumbai, India). The isolates that exhibited typical blue/bluish-green colonies were considered as presumptive ESBL^+^ isolates which were further tested for resistance to indicator cephalosporins, cefotaxime (30 mcg), ceftazidime (30 mcg), cefpodoxime (10 mcg) by disc diffusion test. The resistant isolates were further tested against cefoxitin (30 mcg), imipenem (10 mcg), meropenem (10 mcg), ertapenem (10 mcg), aztreonam (30 mcg), amoxicillin/clavulanic acid (30 mcg), piperacillin/tazobactam (100/10 mcg), and ciprofloxacin (5 mcg). The zones of inhibition were measured and interpreted as resistant or sensitive according to Clinical and Laboratory Standards Institute guidelines [29].

### 2.3. Detection of ESBL Phenotypes

The ESBL production was detected by double disc synergy test [29]. The test bacterium was grown in Mueller Hinton (MH) broth to 0.5 McFarland units and inoculated onto MH agar to form a lawn culture. An amoxicillin/clavulanic acid disc (30/10 µg) was placed at the center of the plate and ceftazidime (30 µg), cefotaxime (30 µg), and cefpodoxime (10 µg) discs were placed 20–30 mm away from the central disc. An extension in the zone of inhibition around the peripheral disc towards the centrally placed amoxicillin/clavulanic acid disc by at least 5 mm indicated ESBL production. The ESBL^+^ phenotype was further confirmed by using Triple ESBL detection Ezy MIC^TM^ Strip (Hi-Media, Mumbai, India) following manufacturer’s instructions. The upper half of the strip (mix^+^) was coated with ceftazidime, cefotaxime, and cefepime plus clavulanic acid; while the lower half (mix) was coated with ceftazidime, cefotaxime, and cefepime coated in a concentration gradient in the reverse direction. The test cultures were prepared as described above and the strips were placed on the culture. The inhibitory concentrations on two sides of the strip were read and a culture was considered ESBL^+^ if the ratio of mix value to mix^+^ value was greater than or equal to 8.

### 2.4. Detection of Resistance Genes by PCR

Amplification of ESBL genes was performed using previously published primers and protocols for *bla*_CTX_ [30], *bla*_SHV_ [31], *bla*_TEM_ [32], and *bla*_NDM_ genes [33,34]. Bacterial DNA was extracted using Wizard DNA kit (Promega, Madison, WI, USA) was used as template for PCR amplification in a Hybaid thermal cycler (Thermo Fisher Scientific Inc., Waltham, MA, USA). For confirmation, representative PCR products were cloned using Strataclone PCR cloning kit (Agilent, Santa Clara, CA, USA) and sequenced (Xcelris Labs, Ahmedabad, India).

## 3. Results

### 3.1. Species Diversity of ESBL^+^ Enterobacteria Isolated from Seafood

From 19 samples of seafood which included 14 finfish and 5 shellfish samples, 215 enterobacterial isolates were obtained of which 169 were ESBL^+^. Of these, 131 (85.62%) isolates were from finfish and 38 (61.29%) were from shellfish. The ESBL^+^ phenotype of the isolates was observed on chromogenic medium followed by confirmation using double disc diffusion and ESBL strip methods. In our study, the chromogenic ESBL agar was found to be a good medium for presumptive isolation of ESBL^+^ enterobacteria from seafood. *E. coli* was the predominant species isolated from seafood, followed by *Klebsiella oxytoca*, *K. pneumoniae*, and *Citrobacter diversus* (Table 1). Nearly two-thirds of all these isolates were ESBL^+^ (Table 1).

### 3.2. Distribution of ESBL^+^ Enterobacteria in Seafood

All samples of fresh fish and shellfish analyzed in our study harbored ESBL^+^ enterobacteria (Table 2). All enterobacterial isolates from finfish such as *Escualosa thoracata*, *Epinephelus diacanthus*, *Terapon jarbua*, *Scomberomorus commerson*, *Otolithes cuvieri*, and shellfish *Acetes indicus* were ESBL^+^.

### 3.3. Antibiotic Resistance of Isolates

Table 3 shows the details of antibiotic resistance of the isolates. Of 169 isolates, 165 (97.63%) were resistant to cefotaxime, 156 (92.30%) to cefpodoxime, 154 (91.12%) to ceftazidime and 109 (65.08%) to aztreonam. Relatively less resistance was seen against imipenem (18 isolates, 10.65%), and ciprofloxacin (27 isolates, 15.97%). Among others, 34 (20.12%) isolates were resistant to cefoxitin, 53 (31.36%) to meropenem, 65 (38.46%) to amoxicillin-clavulanic acid, and 69 (40.82%) to piperacillin/tazobactam. Analysis of the multidrug resistance patterns of enterobacteria showed that 65 (38.5%) isolates were resistant to at least 5–6 antibiotics, 35 (20.71%) isolates to 7–10 antibiotics, 48 (28.4%) isolates to 3–4 antibiotics and 18 (10.6%) isolates to 1–2 antibiotics. Three isolates, *E. coli*, *K. oxytoca*, and *C. diversus* were resistant to all 11 antibiotics tested.

### 3.4. PCR Detection of β-LactamaseGenes

The PCR detected at least one ESBL gene in 161 isolates (Table 4). The *bla*_CTX_ gene was detected in 130 isolates (76.92%), *bla*_SHV_ in 107 isolates (63.31%) and *bla*_TEM_ in 75 (44.37%) isolates, while *bla*_NDM_ was detected in two isolates. Among different species, the occurrence of *bla*_CTX_ was highest in *K. oxytoca* (88.8%), while 73% of the *E. coli* isolates harbored this gene. *bla*_SHV_ gene, on the other hand, was more prevalent in *K. pneumoniae* (73.91%). *bla*_TEM_ was also more prevalent in *K. pneumoniae* (65.21%) (Table 4).

### 3.5. Co-Occurrence of ESBL Genes in Enterobacteria

**Figure 1:**
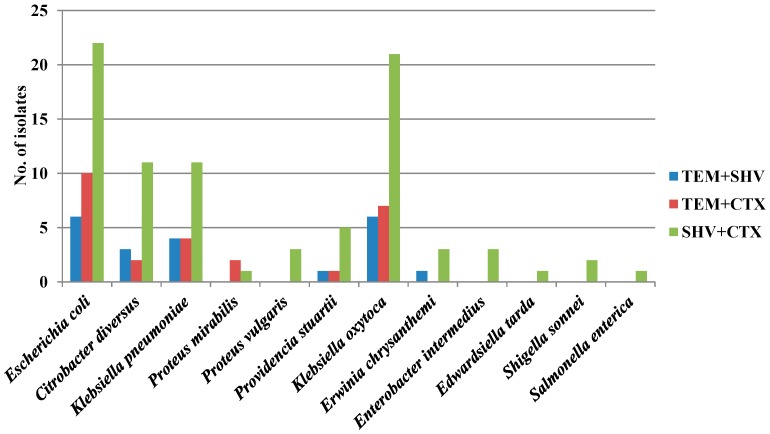
Co-occurrence of two ESBL genes in antibiotic-resistant isolates.

At least two ESBL-encoding genes were detected in all the isolates. The *bla*_SHV_ + *bla*_CTX_ gene combination was found in 84 (49.70%) isolates. The other combinations, *bla*_TEM_ + *bla*_CTX_ and *bla*_TEM_ + *bla*_SHV_, were found in 26 (15.38%) and 21 (12.42%) isolates respectively (Figure 1). *bla*_SHV_ + *bla*_CTX_ combination was more commonly found in *K. oxytoca* (61.76%) and *E. coli* (41.5%). Three ESBL genes (*bla*_SHV_ + *bla*_CTX_ + *bla*_TEM_) were found to co-occur in 17 (10%) isolates. These included *K. oxytoca* (7), *E. coli* (5), *K. pneumoniae* (3), and *C. diversus* (2) (Figure 1).

### 3.6. bla_NDM_-Harboring Enterobacteria in Seafood

The *bla*_NDM_ gene was detected in two isolates, *E. coli* (EC-121) and *C. diversus* (CD-93). These isolates were resistant to all 11 antibiotics tested in this study. Based on sequencing of full length (815 bp) *bla*_NDM_ genes, these isolates were found to be harboring *bla*_NDM-1_ genes.

## 4. Discussion

Fresh seafood sold in retail markets in India are often found contaminated with coliform bacteria which are antibiotic resistant [23], but no studies have focused on ESBL^+^ *Enterobacteriaceae*. Using MacConkey agar, we could isolate ESBL^+^ enterobacteria belonging to diverse species. Analysis of foods of aquatic origin such as fish is expected to yield diverse species of bacteria and therefore, complete biochemical identification to species level is difficult and time consuming. This can be overcome by partial sequencing of 16SrDNA as done in this study, which enabled us to identify 12 species of bacteria belonging to the *Enterobacteriaceae* family (Table 1). Genetic exchange in the aquatic environment among related species leading to acquisition of resistance genes could possibly explain the occurrence of diverse multidrug-resistant enterobacteria in seafood. Our study reports the incidence of ESBL genes in diverse members of *Enterobacteriaceae* family for the first time, although a few past studies have reported the occurrence of antibiotic resistant *E. coli* in seafood. In one study, >80% of *E. coli* isolated from an estuary in Kochi, India, were resistant to multiple antibiotics—such as streptomycin, tetracycline, vancomycin, novobiocin, kanamycin, and oxytetracycline—and these included pathogenic groups such as STEC, ETEC, and EPEC [35]. A recent study [33] has found high levels of ampicillin and ciprofloxacin resistance in *E. coli* strains from seafood in India. Although limited numbers of samples from different fish species were analyzed in our study, the results clearly point to the contamination of seafood with multiple antibiotic-resistant, ESBL^+^ enterobacteria. Among finfish, samples of *Escualosa thoracata*, *Epinephelus diacanthus*, *Terapon jarbua*, *Scomberomorus commerson*, and *Otolithes cuvieri* were positive for ESBL, while a single sample of *Acetes indicus* tested also harbored ESBL^+^ enterobacteria (Table 2). More than 90% of the isolates were resistant to cefotaxime, ceftazidime, and cefpodoxime, while 65.08% of the isolates were aztreonam resistant (Table 3). Among carbapenems, predominant resistance was observed against ertapenem (40.82%), followed by meropenem (31.36%), and less resistance was found against imipenem (10.65%). Carbapenems are advocated for the treatment of infections by ESBL-producing *E. coli* and *K. pneumoniae* [36]. By definition, ESBLs confer resistance to all penicillins, third generation cephalosporins (ceftazidime, cefotaxime, and ceftriaxone) and the monobactams drug aztreonam, but not to cephamycins (cefoxitin and cefotetan) and carbapenems [37]. Carbapenem resistance is mediated by one or more types of carbapenemases [10]. Since the goal of this study was to understand the prevalence of ESBL^+^ enterobacteria, we did not study the mechanisms of carbapenem resistance in our isolates. Furthermore, 15.98% of the isolates were ciprofloxacin-resistant. A study from Korea on commercial seafood has reported that 11.7% of the *E. coli* strains were resistant against cephalothin and 6.7% were resistant against ampicillin, but no resistance was found against amoxicillin/clavulanic acid and cefoxitin [38], while in our study, 38.46% of the isolates were resistant to amoxicillin/clavulanic acid (Table 3). Miranda et al. [39] reported multiple antibiotic-resistant enterobacteria in commercial fish captured from Concepcion Bay, Chile. The unregulated use of antibiotics and release of untreated sewage containing antibiotic residues as well as resistant bacteria from humans and animals result in the emergence and spread of antibiotic-resistant bacteria in the aquatic environment. Interactions among diverse bacteria in the aquatic environment can lead to the exchange of resistance genotypes resulting in the acquisition of multiple antibiotic-resistant mechanisms by human pathogens [40]. Enterobacteria isolated in our study included those resistant to multiple cephalosporins, carbapenems, and even the fluoroquinolone drug, ciprofloxacin. A recent report from the Infectious Diseases Society of America listed ESBL-producing *Klebsiella* spp. and *E. coli* among the most important six drug-resistant microbes to which new therapies are urgently needed [41]. The multidrug resistance to all cephalosporins, fluoroquinolones, and carbapenems leaves little scope to control these MDR bacteria with available antibiotics. A survey of antibiotic resistance in *E. coli* and *K. pneumoniae* over a period of 10 years in India revealed that the ESBL producing enterobacteria have increased from 40% in 2002 to 61% in 2010 [42].

Molecular analysis showed that *bla*_CTX-M_ was the predominant ESBL gene in *Enterobacteriaceae* from seafood, with 76.92% of the isolates being positive for this gene. In our study, 12 species of bacteria were isolated which included diverse members of the family *Enterobacteriaceae* (Table 4). Among these, *K. oxytoca* (88.8%) was predominantly ESBL^+^ followed by *E. coli* (73%). *E. coli* and *K. pneumoniae* are the most common enterobacteria harboring TEM-type β-lactamases, but their occurrence in other bacterial species is increasingly being reported. Some of these include *Enterobacter aerogenes*, *Morganella morganii*, *Proteus mirabilis*, *Proteus rettgeri*, and *Salmonella enterica* [43]. In *E. coli*, *bla*_ctx_ are often found on the plasmids which increases their chances of horizontal dissemination [44]. Plasmid transfer is supposedly a common event in the aquatic environment leading to rapid spread of resistance genotypes [45].

All the isolates harbored two or more ESBL genes (Table 4). Several studies on the isolation of ESBL^+^ bacteria from food animals have also reported the simultaneous occurrence of more than one ESBL genes. TEM, SHV, CTX-M-producing organisms have been detected in a variety of food-producing animals (poultry, swine, bovine, horse, rabbit, ostrich, wild boars) and foods of animal origin [46]. However, not many studies are available on seafood isolates. Ryu et al. [38] detected *bla*_TEM_ gene in 21.4% of the isolates which were predominantly ampicillin-resistant. *bla*_TEM_, but not other ESBL genes, have been reported in shellfish isolates of *E. coli* from Vietnam [47]. Our study reports for the first time, the occurrence of multiple ESBL genes in seafood isolates of enterobacteria.

A significant percentage of isolates in this study were resistant to carbapenems (Table 3). Diverse enzymes belonging to the metallo-β-lactamase (MBL) family confer resistance to carbapenems, cephalosporins, and penicillins [48]. MBLs are mainly of the IMP, VIM, SPM, GIM, SIM, and NDM types, with IMP, VIM, and NDM being the most prevalent enzymes [49]. The New Delhi Metallo-β-Lactamase (*bla*_NDM_) is the most recent, initially reported from India in *Klebsiella pneumoniae* and *Escherichia coli* [10], and later from different countries in diverse species of Gram-negative bacteria [50]. We recently reported the isolation of *bla*_NDM_-harboring *E. coli* in seafood in Mumbai, India. In this context, we screened all ESBL^+^ isolates for the presence of *bla*_NDM_ gene and found two isolates to be *bla*_NDM_-positive. Of these, EC-121 was isolated from fish *Otolithes cuvieri*, while CD93 was isolated from clam species *Meretrix meretrix*. Both the isolates were resistant to all the antibiotics tested in this study (data not shown) and also harbored multiple ESBL genes. EC-121 carried *bla*_CTX-M_, *bla*_SHV_, and *bla*_TEM_ genes, whereas CD-93 harbored *bla*_TEM_ and *bla*_SHV_ genes. The incidence of *bla*_NDM_-harboring bacteria has not been reported in seafood from other countries. However, NDM-positive bacteria are increasingly being reported from aquatic ecosystems raising concerns of such bacteria getting established in the environment and disseminated via foods [40]. *bla*_NDM-1_-positive *K. pneumoniae* has been reported from Kim Nguu River of Vietnam [13]. *Acinetobacter baumannii* isolated from the sewage of hospitals in Beijing, China reportedly harbored *bla*_NDM-1_ [14]. The isolation of *bla*_NDM-1_-positive enterobacteria from clinical settings has been reported from Mumbai [51,52]. However, studies are necessary to determine if *bla*_NDM-1_ bacteria in the environment are derived from clinical sources or if other reservoirs, such as animals and birds also exist. The use of antibiotics in animal husbandry may also contribute to the dissemination of such bacteria into the environment. In isolates which were carbapenem resistant but *bla*_NDM_ negative, other MBL genes may be responsible for the resistance, although this was not investigated further. The plasmid-mediated MBL genes have wider dissemination ability and rapid acquisition of such genes by pathogenic bacteria in the aquatic environment can lead to their rapid spread in community via food and water. 

The high incidence of ESBL-positive enterobacteria observed in this study not only suggests a health risk, but also implicates seafood as vehicles of their dissemination into the households. It is important to identify the critical points of contamination of seafood with antibiotic-resistant bacteria. Strategies such as scientific management of domestic sewage, regulation of human settlement, and pollution along the coast and development of hygienic market facilities for seafood are needed to ensure the quality and safety of seafood.

## Figures and Tables

**Table 1:** Species composition of ESBL^+^ enterobacteria from seafood.

Species	Number Isolated (ESBL^+^)
*Escherichia coli*	66 (53)
*Klebsiella oxytoca*	34 (27)
*Klebsiella pneumoniae*	30 (23)
*Citrobacter diversus*	28 (22)
*Erwinia chrysanthemi*	16 (12)
*Proteus vulgaris*	13 (10)
*Proteus mirabilis*	8 (6)
*Providencia stuartii*	8 (6)
*Enterobacter intermedius*	7 (5)
*Edwardsiella tarda*	2 (2)
*Shigella sonnei*	2 (2)
*Salmonella enterica*	1 (1)
Total	215 (169)

**Table 2:** Details of ESBL^+^ enterobacteria isolated from different seafood types.

Sample Types (Number Analyzed)	No. of Enterobacteria Isolated	No. (%) of ESBL^+^ Enterobacteria
**Fish (14)**		
*Sardinella albella* (5)	41	31 (75.60)
*Coilia dussumieri* (3)	39	29 (74.35)
*Escualosa thoracata* (1)	14	14 (100)
*Epinephelus diacanthus* (1)	15	15 (100)
*Harpadon nehereus* (1)	11	9 (81.81)
*Terapon jarbua* (1)	7	7 (100)
*Scomberomorus commerson* (1)	10	10 (100)
*Otolithes cuvieri* (1)	16	16 (100)
**Shrimp (3)**		
*Acetes indicus* (1)	12	12(100)
*Metapenaeus dobsonii* (2)	19	14 (73)
**Clam (1)**		
*Meretrix meretrix*	16	8 (50)
**Squid (1)**		
*Loligo duvauceli*	15	4 (26)
**Total**	**215**	**169**

**Table 3:** Antimicrobial susceptibility profiles of ESBL^+^ isolates.

Antibiotics Tested	No. (%) Resistant ^a^
Cefoxitin (CX)	34 (20.12%)
Cefotaxime (CTX)	165 (97.63%)
Ceftazidime (CAZ)	154 (91.12%)
Cefpodoxime (CPD)	156 (92.30%)
Imipenem (IPM)	18 (10.65%)
Ertapenem (ETP)	69 (40.82%)
Meropenem (MRP)	53 (31.36%)
Ciprofloxacin (CIP)	27 (15.98%)
Aztreonam (AT)	110 (65.08%)
Amox/clav (AMC)	65 (38.46%)
Piperacillin/Tazobactam (TZP)	69 (40.82%)

^a^ Total number of isolates tested = 169.

**Table 4:** Distribution patterns of ESBL genes in different species of enterobacteria.

Species	Number of Isolates Tested	Distribution of ESBL Genes		
		*bla*_CTX_	*bla*_SHV_	*bla*_TEM_
*Escherichia coli*	53	41	29	24
*Klebsiella oxytoca*	27	24	24	16
*Klebsiella pneumoniae*	23	15	17	15
*Citrobacter diversus*	22	15	11	8
*Erwinia chrysanthemi*	12	6	7	6
*Proteus vulgaris*	10	9	3	0
*Proteus mirabilis*	6	6	1	3
*Providencia stuartii*	6	6	6	3
*Enterobacter intermedius*	5	3	4	0
*Edwardsiella tarda*	2	2	2	0
*Shigella sonnei*	2	2	2	0
*Salmonella enterica*	1	1	1	0
**Total**	**169**	**130**	**107**	**75**
